# A new efficient method for analyzing fungi species using correlations between nucleotides

**DOI:** 10.1186/s12862-018-1330-y

**Published:** 2018-12-27

**Authors:** Xin Zhao, Kun Tian, Stephen S.-T. Yau

**Affiliations:** Department of Mathematical Sciences, Tsinghua University, Beijing 100084, People’s Republic of China

**Keywords:** DNA barcoding, Fungi species, Classification, Correlations between nucleotides, 18-dimensional natural vector, Phylogenetic analysis

## Abstract

**Background:**

In recent years, DNA barcoding has become an important tool for biologists to identify species and understand their natural biodiversity. The complexity of barcode data makes it difficult to analyze quickly and effectively. Manual classification of this data cannot keep up to the rate of increase of available data.

**Results:**

In this study, we propose a new method for DNA barcode classification based on the distribution of nucleotides within the sequence. By adding the covariance of nucleotides to the original natural vector, this augmented 18-dimensional natural vector makes good use of the available information in the DNA sequence. The accurate classification results we obtained demonstrate that this new 18-dimensional natural vector method, together with the random forest classifier algorthm, can serve as a computationally efficient identification tool for DNA barcodes. We performed phylogenetic analysis on the genus *Megacollybia* to validate our method. We also studied how effective our method was in determining the genetic distance within and between species in our barcoding dataset.

**Conclusions:**

The classification performs well on the fungi barcode dataset with high and robust accuracy. The reasonable phylogenetic trees we obtained further validate our methods. This method is alignment-free and does not depend on any model assumption, and it will become a powerful tool for classification and evolutionary analysis.

**Electronic supplementary material:**

The online version of this article (10.1186/s12862-018-1330-y) contains supplementary material, which is available to authorized users.

## Background

The identification and phylogenetic analysis of living species are crucial tasks for understanding natural biodiversity. Conventional taxonomy based on morphological and ecological data is often challenging work. Not only does it require a highly experienced taxonomist, but also it is usually quite time consuming. Moreover, previous studies have revealed that traditional phenotypic recognition of taxonomy may lead to misidentification [1]. Advancements in sequencing and computational technologies enable the production of a large number of DNA sequences in a very short time. DNA genomes contain all genetic material of organisms, and are becoming the major source of new information for our understanding in evolutionary relationships [2]. However, the complexity and large size of genomes make it inappropriate for distinguishing species rapidly. In the last two decades, researchers have proposed using the information from one or a few gene regions, termed DNA barcodes, to easily discriminate species [1].

A DNA barcode is a standardized short fragment (500–800 bp) of a DNA sequence that characterizes and identifies the species of a specimen [3]. The gene region must satisfy some properties. For example, the fragment must contain significant species-level genetic diversity. Specimens of the same species should have identical fragments, but the fragments from specimens of different species should differ. In addition, the fragment must be adaptable and conserved with primer binding sites allowing it to be readily for PCR amplification [4]. Several studies have established and described the significance of this approach for taxonomic work. DNA barcoding has been developed with great success for identifying groups of living species, including animal [5], plants [6], fungi [3], bacteria [7] and algea [8]. Consequently, combining DNA barcoding with traditional taxonomic tools could accurately and effectively reveal biodiversity. Although researchers are trying to find a suitable biomarker for the discrimination of all taxa, a universal DNA barcode has not been found. The region of mitochondrial gene cytochrome c oxidase (COI) [9] can serve as a DNA barcode for most animal groups. The internal transcribed spacer (ITS) region has been recommended as the most appropriate DNA marker for barcoding universal fungi [3, 10].

Global online workbenches have established databases for DNA barcode records, such as the Consortium for the Barcode of Life (CBOL, http://barcoding.si.edu) and the Barcode of Life Data System (BOLD, http://www.barcodinglife.org). Meanwhile, these databases are freely available to researchers interested in DNA barcoding. By comparing a DNA barcode sequence obtained from an unidentified specimen with sequences from known species in the reference database, we can determine the species or group of the new specimen. In March 2018, the BOLD [11] contained more than 6 million specimens with barcode records, belonging to over 277,013 species. This situation encourages us to determine the reliable assignment in an accurate and fast manner.

Traditional barcoding methods are usually performed by classical phylogenetic approaches, such as neighbor-joining [12] and maximum parsimony [13]. Some statistical models for data analysis have also been proposed, such as sophisticated Bayesian [14], decision theory [15] and some other approaches [16, 17]. These methods have greatly contributed to DNA barcoding research. However, a number of challenges remain, including proper choice of the threshold and computational efficiency, as well as the accuracy of classification. In this study, we investigate a new alignment-free method for DNA barcoding. Our focus is on performing this assignment task accurately and efficiently.

In this work, we develop a new representation for charactering DNA sequence which is based on the distribution on nucleotides within the sequence. To accomplish this, we add the covariance of nucleotides to the original natural vector. As a result, the new 18-dimensional natural vector makes good use of the available information in the sequence. We used the fungi barcode dataset as the test dataset, because we want to improve the relatively low accuracy of fungi barcode classification in the previous studies [3, 18]. We also analyzed and evaluated the genetic distance within and between species of barcoding dataset. On one hand, the one-to-one correspondence between DNA barcodes and their 18-dimensional vectors ensures the barcoding sequence information is not lost. On the other hand, because the difference between intraspecific variation and interspecific variation is reflected in vector distances, this method shows promise for it being used to distinguish species and identify specimens into correct species with higher accuracy and less time. Furthermore, we also investigated the phylogenetic relationship between species using fungi DNA barcode sequences.

## Results

### Convex hull analysis of DNA barcodes

**Fig. 1:**
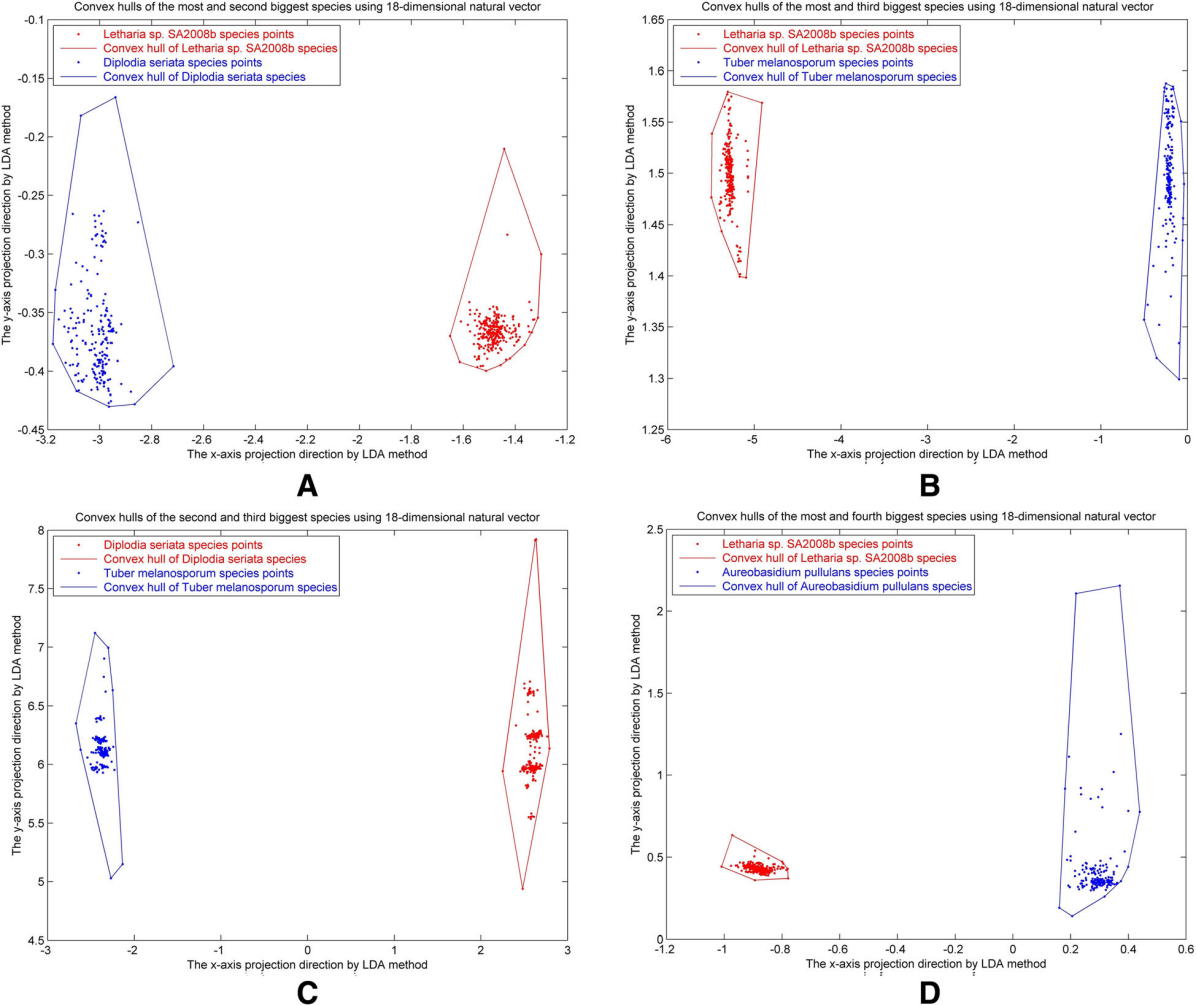
Convex hulls of pairs of species after dimension reduction by LDA method. In each of figures (**a**, **b**, **c** and **d**), the convex hulls of the species (represented by red points) have no intersection with those represented by the blue points. **a** Red points: *Letharia sp. SA2008b* (260 points), blue points: *Diplodia seriata* (204 points). **b** Red points: *Letharia sp. SA2008b* (260 points), blue points: *Tuber melanosporum* (189 points). **c** Red points: *Diplodia seriata* (204 points), blue points: *Tuber melanosporum* (189 points). **d** Red points: *Letharia sp. SA2008b* (260 points), blue points: *Aureobasidium pullulans* (167 points)

The DNA barcodes dataset used in this study consisted of 72844 barcode sequences from 25278 species. The number of available barcode specimens differs greatly across species. Please see the Methods section for further details. For each barcode specimen, we first calculated the 18-dimensional natural vector to describe the distribution of the four nucleotides within the barcode. Then, for each fungi species we constructed the convex hull in 18-dimensional space using the vectors corresponding to the barcode specimens belonging to that species. An analysis using linear programming analysis showed there no two pairs of convex hulls intersected. Our results indicate that barcode sequences with similar distributions of the four nucleotides should be in the same species. The results are also consistent with the central law of molecular biology. In order to visualize the results, we applied the linear discriminant analysis (LDA) method for dimension reduction. LDA is a method used to determine whether two groups are linearly separable. The dimension of the natural vectors was reduced from 18 to 2. We used the species which contain the four largest number of barcode specimens to demonstrate this property. Projections of the convex hulls for these four species are shown in Fig. 1. We can clearly see that the points representing the ITS region of the same species in genome space are clustered, rather than being broadly distributed. This suggests that as new barcode specimens are included, their points will lie approximately within the convex hull of the points corresponding to known species.

### Classification performance

**Fig. 2:**
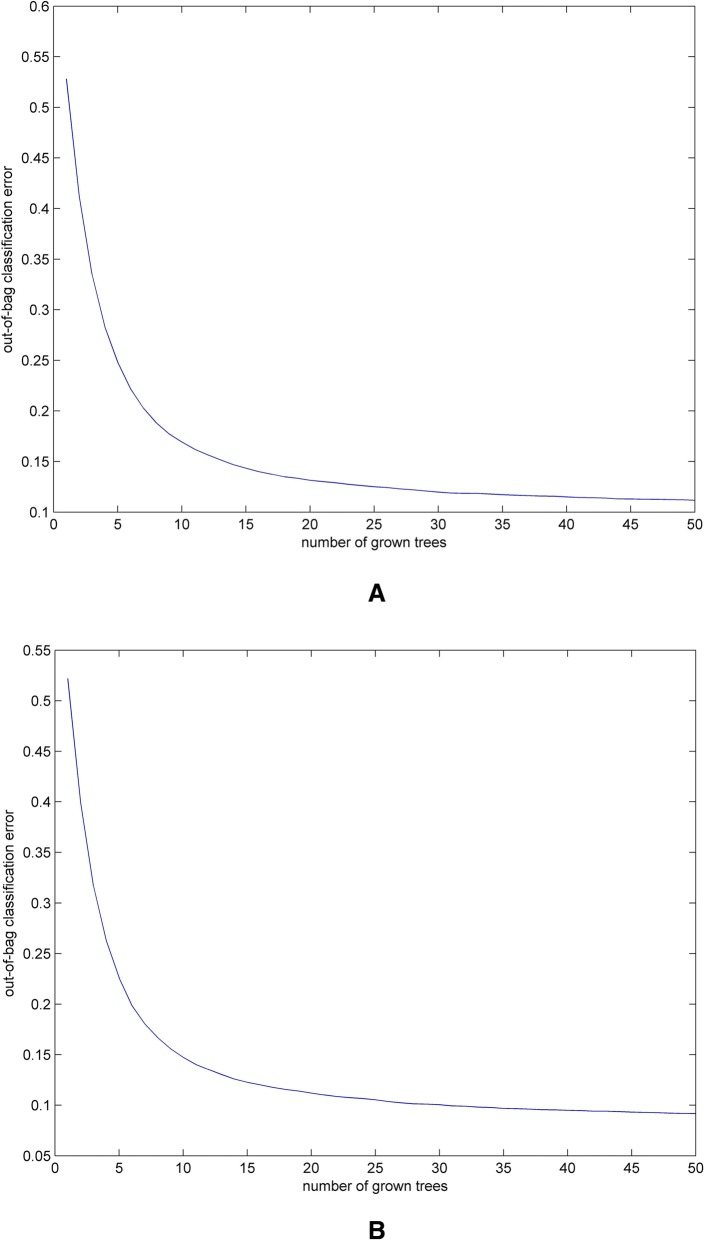
Out-of-bag error for the bagging algorithm on two datasets used in this study. **a** Out-of-bag error with the increased number of sample trees on 72247 barcode sequences dataset. **b** Out-of-bag error with the increased number of sample trees on 56392 barcode sequences dataset

**Fig. 3:**
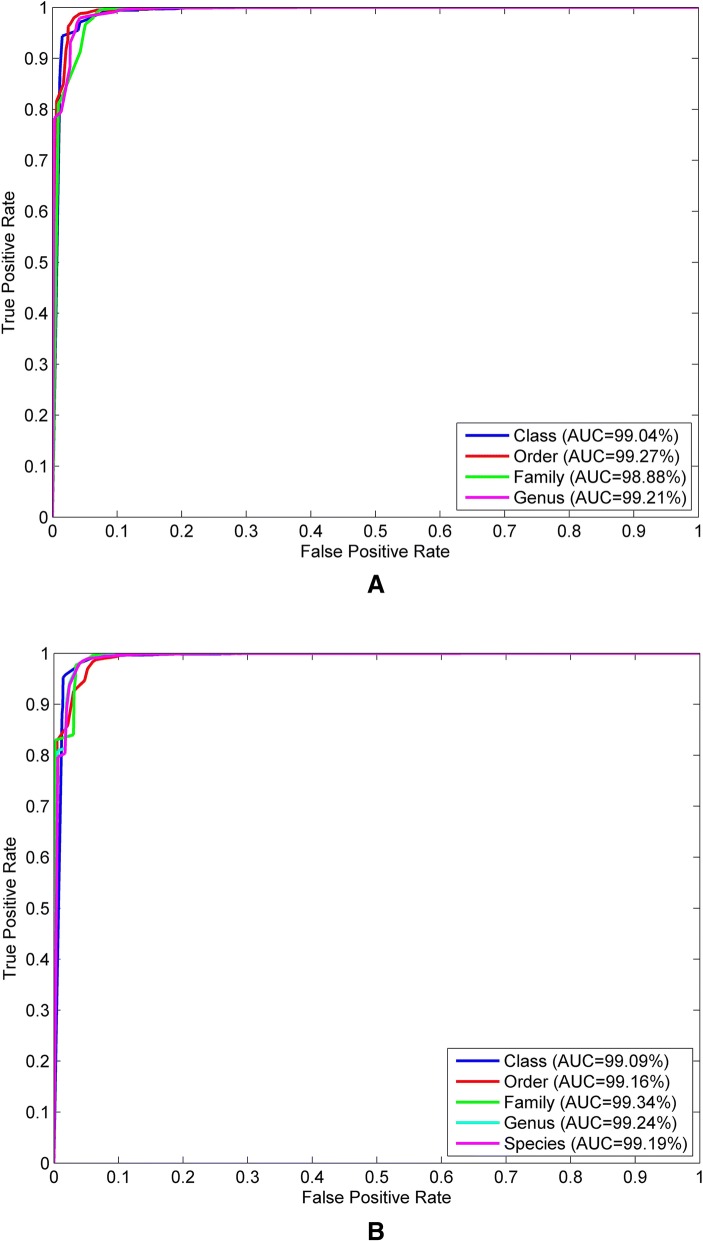
ROC curves for the classification results on two datasets used in this study. **a** ROC curves for the classification results of class, order, family and genus on 72247 barcode sequences dataset. **b** ROC curves for the classification results of class, order, family, genus and species on 56392 barcode sequences dataset

We examined the classification performance of the 18-dimensional natural vectors on barcode sequences. After filtering the taxonomy information, we reserved the sequences with completed information containing class, order, family, genus as well as species taxonomy. There are 72247 barcode sequences sourced from 24681 species, which could be divided into 1740 genera with more than one species for each genus. Further detailed information can be found in the Methods section. The random forest algorithm was used to classify the sequences into the four taxonomic groups including class, order, family, and genus. The accuracy of classification was tested by predicting the barcodes in the dataset and comparing against their existing classification. The reason we choose this classifier is that we could get an unbiased estimate of the error during the generation process instead of using another independent test dataset. According to bagging algorithm, the optimal number of trees could be observed by the out-of-bag error. Figure 2a shows the out-of-bag error with the increased number of trees. We could observe the training and test error quickly decrease with five sample trees and tend to level off after twenty trees have been fit. Thus, we used both five and twenty sample trees for this classification. The accuracy of five sample trees for class, order, family and genus was 98.6, 98.27, 98.07 and 97.73%, respectively. The classification results were more accurate with twenty sample trees. The average accuracy for class, order, family and genus was 99.3, 99.96, 99.97 and 99.96%, respectively. On the other hand, we drew the ROC curves for the classification results of class, order, family and genus shown in Fig. 3a. The detailed steps for ROC curves can be found in Methods section. In this figure, we can see the areas under ROC curves represented by results obtained using the random forest algorithm achieve more than 0.98.

Although the distribution of barcodes specimens for different species is uneven, we want to explore the performance of identifying barcode sequences into species using natural vectors. After recognizing and removing the species with only one barcode, we were left with 56392 sequences from the original dataset. The 56392 barcodes belong to 8826 species and 1465 genera, as well as 349 families, 107 orders and 30 classes. We evaluated the assignment in the similar way as described above. The out-of-bag error calculated in this dataset is shown in Fig. 2b. We selected five and twenty sample trees to compute the identification. The prediction accuracy was 96.87 and 99.91% respectively for species assignment with different number (five and twenty) of sample trees. In addition, we studied the efficacy at higher taxonomic levels. The calculated error rates of higher taxonomic groups are about 0.2–0.5% using random forest classifier with twenty sample trees. We also drew the ROC curves for the classification results of class, order, family, genus and species shown in Fig. 3b. In this figure, the areas under ROC curves achieve more than 0.99. Consequently, these results indicate the ITS region is a suitable barcode for species and also for higher taxonomic levels. Moreover, our classification success demonstrates that the new 18-dimensional natural vector representation method together with the random forest classifier algorithm can serve as an effective identification tool for DNA barcodes.

### Genetic distance and statistical analysis

**Fig. 4:**
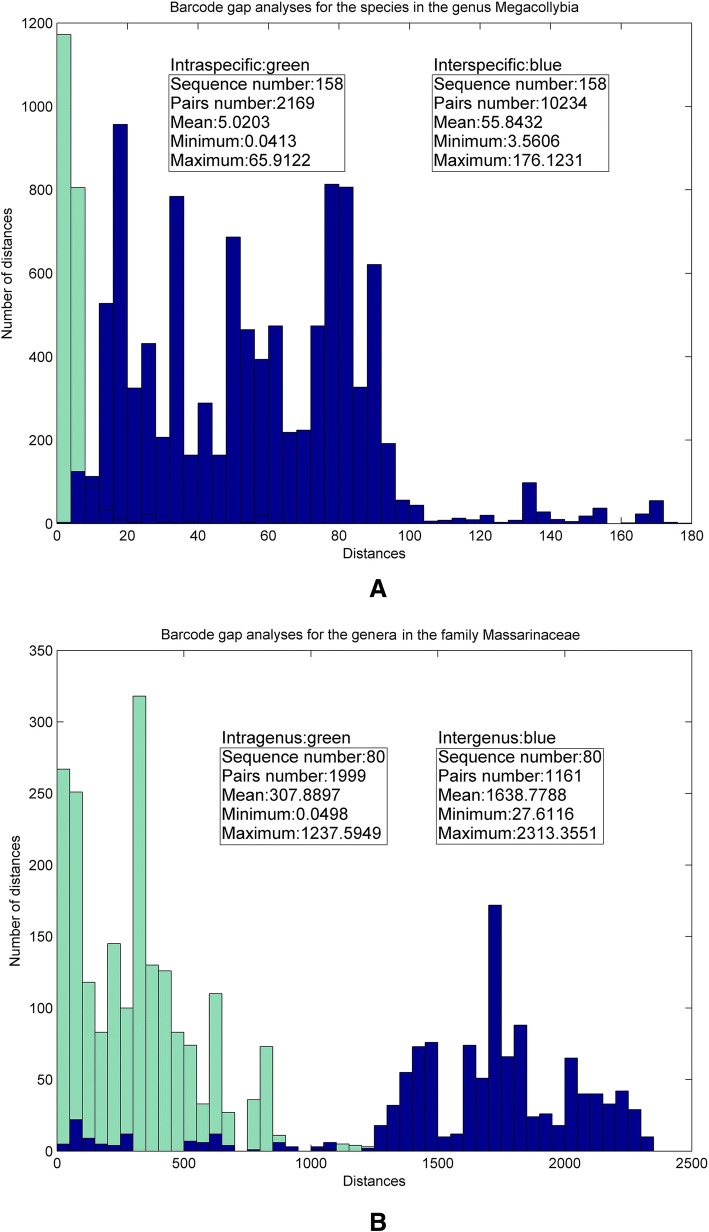
Barcode gap analyses for the genus *Megacollybia* and the family *Massarinaceae* using distance histograms. **a** Histograms display the intraspecific variation in green and the interspecific variation in blue for the genus *Megacollybia*. **b** Histograms display the intragenus variation in green and the intergenus variation in blue for the family *Massarinaceae*

We next checked whether the new natural vector representation reflects differences in genetic distance. One significant property of barcodes is the barcode gap [3]. The interspecific variation (between species) should be clearly and preferably significantly greater than the intraspecific variation (within species). We performed analyses using genus *Megacollybia* and family *Massarinaceae* as examples. After computing 18-dimensional natural vectors for each species in the genus *Megacollybia*, the Euclidean distance was used to calculate intraspecific differences, as well as interspecific differences. A graphical representation using a histogram of distance distribution results is shown in Fig. 4a. On one hand, we could observe the clear gap between mean intraspecific distance (5.02) and mean interspecific distance (55.84). On the other hand, intraspecific distances of most species are much smaller than the mean interspecific distance and vice versa. In addition to this, we also studied the distance variability at the genus level. The significant barcode gap between intragenus distance and intergenus distance that was found, is shown in Fig. 4b. As we can see from Fig. 4b, most of intergenus distances are much larger than the mean intragenus distance (307.89). The genus *Megacollybia* and family *Massarinaceae* are the two examples which show the barcode gap most clearly. We have added Additional file 1: Figures S1-S6 to the additional file which show more barcode gap examples. These analyses confirm the potential of the ITS region for barcoding fungi on different taxonomic levels.

### Phylogenetic analysis on DNA barcodes for species

**Fig. 5:**
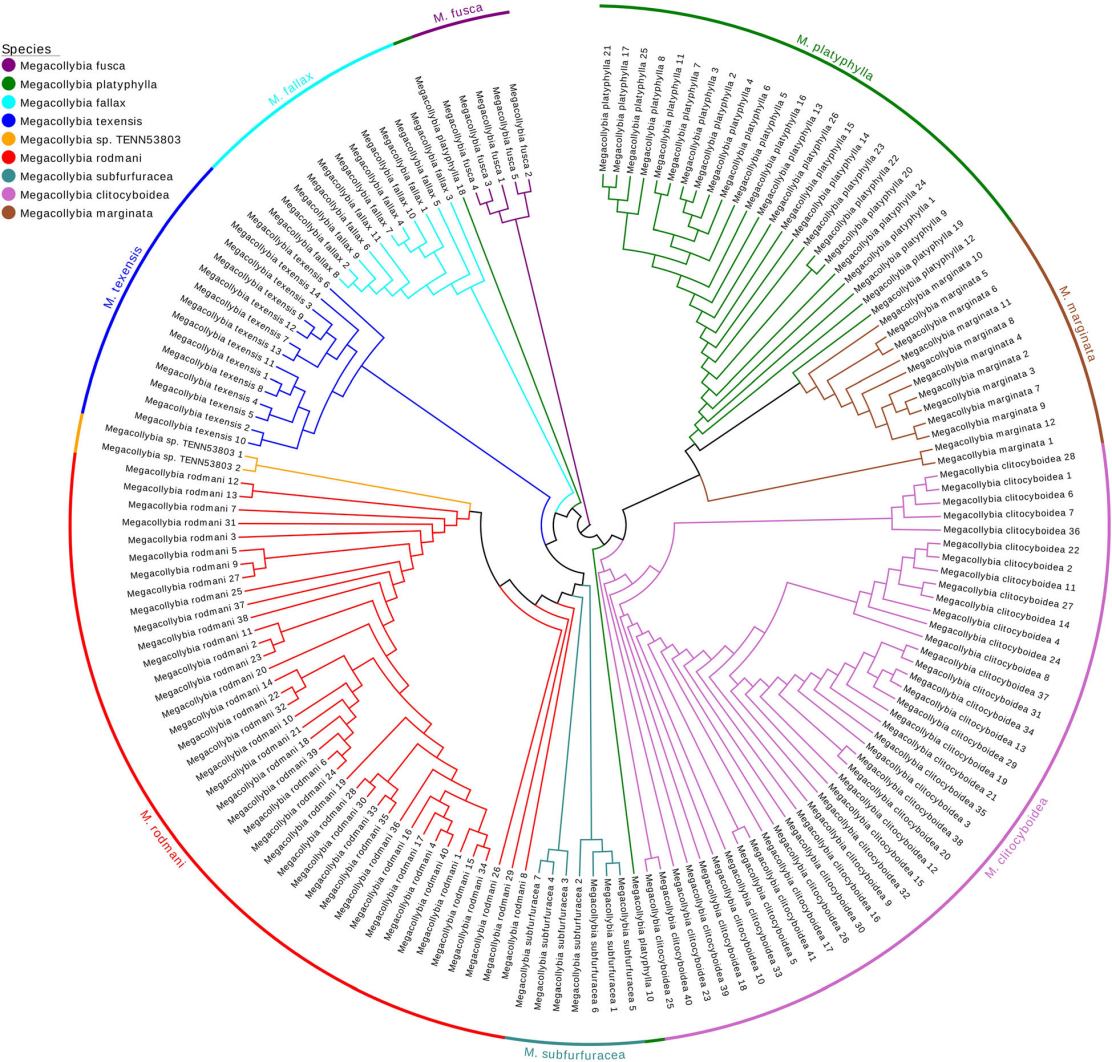
Phylogenetic tree for the genus *Megacollybia*. The tree was constructed using single linkage algorithm with the 18-dimensional natural vector method. Different colors were allocated to represent different species

**Fig. 6:**
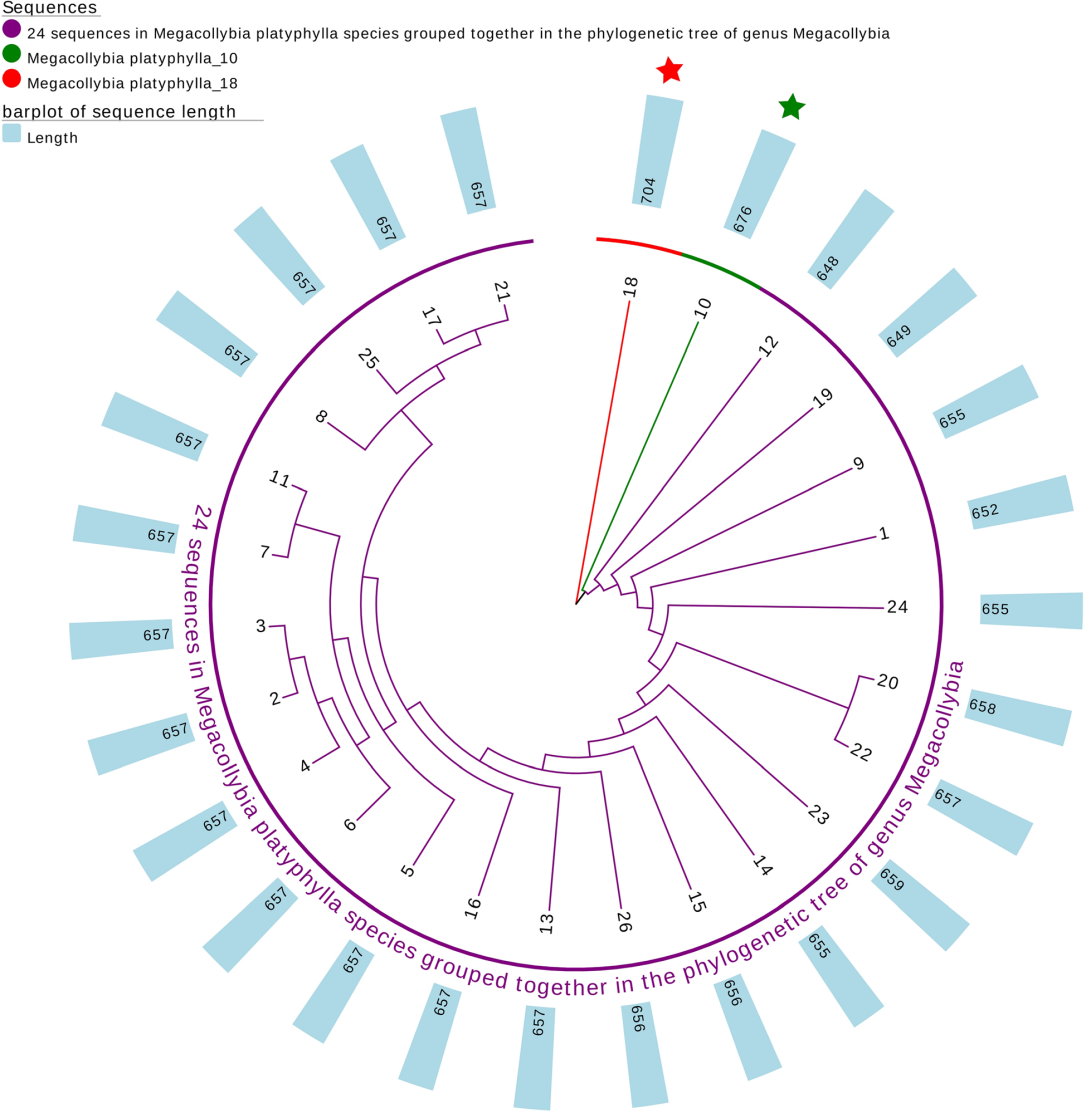
Phylogenetic tree for the species *Megacollybia platyphylla*. The tree was constructed using single linkage algorithm with the 18-dimensional natural vector method. The two special sequences are marked with red and green, and the lengths of barcode sequences are also shown

Furthermore, we performed phylogenetic analysis using DNA barcodes to demonstrate the validation of our method. For the above genus *Megacollybia*, we computed the Euclidean Distance between pairs of sequences after calculating the 18-dimensional natural vector for each barcode. We then performed the single linkage algorithm [19] to reconstruct the phylogeny shown in Fig. 5. The phylogeny for sequences within the same species is shown in the same color. From the phylogenetic tree, we can see that nearly all the sequences from the same species cluster together to the same clades, except for two sequences from *Megacollybia platyphylla*. The two sequences *Megacollybia platyphylla 10* and *Megacollybia platyphylla 18* do not group with the other barcode specimens in *Megacollybia platyphylla*. To better explore and understand this situation, we reconstructed the evolutionary relationship of the 26 barcodes from *Megacollybia platyphylla* as shown in Fig. 6. We found that the two sequences are at the basis of other 24 barcodes, and we marked them with stars. We calculated the length and GC content of the 26 barcode sequences. The length of the other 24 barcodes are around 657. The other two sequences are significant longer than them. In addition, the two sequences also have higher GC content than the other. This may the reason that the two barcodes are not in the same clade as the other sequences in the phylogenetic tree. Overall, the reasonableness of the phylogenetic trees confirms that our methods applied on this dataset are convincing.

## Discussion

### Comparison with other methods

We applied the 18-dimensional and 12-dimensional natural vector methods on the genus *Pachyphloeus* consisting of 12 species in the barcode dataset. In this genus, there are 8 species containing only one sequence and the other 4 species are comprised of 2, 3, 10, 13 sequences, respectively. The phylogenetic trees constructed by the 12-dimensional natural vector and 18-dimensional natural vector methods are shown in Additional file 1: Figure S7 and Figure S8.

In Additional file 1: Figure S7, the three sequences in species *Pachyphloeus marroninus* colored orange are not clustered together. Sequence *Pachyphloeus marroninus 3* is wrongly clustered closer to *Pachyphloeus melanoxanthus 2* than the other two sequences in the species *Pachyphloeus marroninus*. By contrast, using the 18-dimensional natural vector method, we find that *Pachyphloeus marroninus 3* is classified near the other sequences belonging to the same species shown in Additional file 1: Figure S8. This shows that the 18-dimensional natural vector method performs better than the 12-dimensional natural vector method in phylogenetic analysis.

On the other hand, we also tested the commonly used multiple sequence alignment method and the k-mer method on the same genus. The phylogenetic trees are shown in Additional file 1: Figures S9 and S10. For the largest species *Pachyphloeus sp.*, neither multiple sequence alignment nor the k-mer method performed well because the barcodes from this species do not cluster together and instead cluster with other species’ barcodes. We marked the wrongly clustered sequences with stars in Additional file 1: Figure S9 and S10. Comparing with Additional file 1: Figure S8, we can see that the results of our 18-dimensional natural vector method outperform that two methods. In addition, the 18-dimensional natural vector method is alignment-free and the computation time is much shorter than multiple alignment method. In conclusion, the new natural method can perform phylogenetic analysis more precisely and quickly.

### Significance for the four features in 18-dimensional natural vector

The 18-dimensional natural vector of a sequence contains four kinds of features including the numbers, the mean positions and the normalized variations of the four kinds of nucleotides as well as the covariance between different nucleotides. The four features are significant and integral of the 18-dimensional natural vector. In order to check which features are more important, we carried out the following process. First of all, we tried deleting one of the four features of 18-dimensional natural vector. We then used the other three features to represent the sequence and perform phylogenetic analysis on the same dataset. The four trees are shown in Additional file 1: Figure S11-S14 in the additional file. By comparing these four trees with that of 18-dimensional natural vector method (Additional file 1: Figure S8), we analyzed the importance of each feature. Among these four trees below, the first and second trees differ more from the tree constructed using the 18-dimensional natural vector than the third and fourth trees. We marked the mixed-up clades with stars in phylogenetic trees shown in Additional file 1: Figure S11 and S12. Although the clades in Additional file 1: Figure S13 and S14 are not as good with that of Additional file 1: Figure S8, these two trees are better than the tress in Additional file 1: Figure S11 and S12. This implies that the number and mean position features are more important than the normalized variation and covariance features. On the other hand, the orders of magnitudes of number and mean position features are about 10^2, while the normalized variation and covariance features are about 10^1.This makes the number and mean position features become dominant in the Euclidean distance between vectors in this study.

## Conclusions

In this study, we present a new method for DNA barcode classification based on the distribution on nucleotides. We consider the statistical information containing the numbers, mean positions, moments and correlations between nucleotides. These features are used to characterize a DNA sequence, forming an 18-dimensional natural vector. Our method has the following main advantages: (1) It contains nearly all important information within a sequence. (2) The mapping between a DNA barcode and its 18-dimensional vector is one-to-one. (3) The covariance measures the correlations between the four nucleotides. We treat the Euclidean distance between the vectors as the similarity metric. We then examine the classification performance for class, order, family, genus as well as species taxonomy by applying the 18-dimensional natural vector method to barcode sequences. Furthermore, further tests on barcode gap analysis and phylogenetic analysis are used to validate the method. The highly accurate results and computationally efficient algorithm provide us a new quantitative way of identifying and analyzing evolutionary relationships among species based on DNA barcodes in molecular biological study.

## Methods

### Datasets

The dataset used in this study is downloaded from the Barcode of Life Data System (BOLD, http://www.barcodinglife.org). The origin dataset contains a total of 88,650 barcodes from 28,058 fungi species. We remove some sequences without fully taxonomic information and preserve the dataset pertaining to ITS region of fungi. There are 72,844 barcodes sourced from 25,278 different species with completed taxonomy information would be analyzed in this study. The current classification scheme for fungi is used in this dataset, in which the taxonomic classification could be split into 38 classes, 135 orders, 448 families and 2337 genera. We first verified the convex hull principle by analyzing these 72,844 barcodes. The distribution of sequences into different species is uneven. Among these 2327 genera, 597 genera have only one barcode member. Except for these 597 sequences, the other 72,247 sequences belong to 24,681 species, 1740 genera, 382 families, 117 orders and 33 classes, which are used for further classification analysis in this study.

### Features of natural vector

Let *S* = (*s*_1_, *s*_2_, *s*_3_, ⋯, *s*_*N*_) be a nucleotide sequence of length *N*, where *s*_*i*_ ∈ {*A*, *C*, *G*, *T*}, *i* = 1, 2, 3, ⋯*N.*

Firstly, we define indicator functions for four nucleotides. For a nucleotide *k*, define

$$ {w}_k\left({s}_i\right)=\left\{\begin{array}{c}1, if\ k\  appear s\  at\  the\  ith\  position\ of\ the\ sequence.\\ {}0, if\ k\  does{n}^{\prime }t\  appear\  at\  the\  ith\  position\ of\ the\ sequence.\end{array}\right. $$

We then calculate three features *n*_*k*_, *μ*_*k*_ and ${D}_2^k$ to describe the number of nucleotide *k*, and the mean position of nucleotide *k* as well as the normalized variation of the position for nucleotide *k* appearing in sequence *S*, respectively. The features are defined as follows:

$$ {n}_k={\sum}_{i=1}^N{w}_k\left({s}_i\right),{\mu}_k=\frac{\sum_{i=1}^Ni\bullet {w}_k\left({s}_i\right)}{n_k},{D}_2^k={\sum}_{i=1}^N\frac{{\left(i-{\mu}_k\right)}^2{w}_k\left({s}_i\right)}{n_kN}, $$

where *k* represents the four nucleotides.

### Covariance between nucleotides

For two finite point sets: *A* = {*a*_1_, *a*_2_, …, *a*_*n*_}, *B* = {*b*_1_, *b*_2_, …, *b*_*m*_} in *R*,which satisfy *a*_1_ < *a*_2_ < … < *a*_*n*_ and *b*_1_ < *b*_2_ < … < *b*_*m*_, the covariance of the two sets *A* and *B* can be calculated in two cases. If *m* = *n*, we define

$$ Cov\left(A,B\right)={\sum}_{i=1}^n\left({a}_i-{\mu}_A\right)\left({b}_i-{\mu}_B\right)/n, $$

where ${\mu}_A={\sum}_{i=1}^n{a}_i/n,{\mu}_B={\sum}_{i=1}^m{b}_i/m.$ If *m* ≠ *n*, assume that *m* > *n*. Then the covariance between *A* and any *n* values in *B* is computed and take the average of these ${C}_m^n$ results as the final covariance *Cov*(*A*, *B*) between the two point sets.

We could compute the covariance of any pair of nucleotides *k*_1_ and *k*_2_ for a sequence *S* of length *N*. Assume that position of *k*_1_ appeared in the sequence *S* is *A* = {*a*_1_, *a*_2_, ⋯, *a*_*n*_}, the position of *k*_2_ is *B* = {*b*_1_, *b*_2_, ⋯, *b*_*m*_}. Then the covariance formula of *k*_1_ and *k*_2_ is defined as

$$ Cov\left({k}_1,{k}_2\right)= Cov\left(A,B\right)/N. $$

It is obvious that when *k*_1_ = *k*_2_, the corresponding formula should be the

$$ Cov\left({k}_1,{k}_2\right)={\sum}_{i=1}^n\frac{\left({a}_i-{\mu}_A\right)\left({a}_i-{\mu}_A\right)}{n\ast N}=\sum \limits_{i=1}^n\frac{{\left({a}_i-{\mu}_A\right)}^2}{n\ast N}={D}_2^{k_1}. $$

The formula above reflects the variance of the position of nucleotides.

We consider the sequence *S* = *ACGTAC* as an example. Based on *μ*_*A*_ = 3, *μ*_*C*_ = 4 and the positions of amino acids *A* = {1, 5} and *C* = {2, 6} in the sequence, we get *Cov*(*A*, *C*) = [(1 − 3)(2 − 4)/2 + (5 − 3)(6 − 4)/2]/6 = 2/3. The other covariance could also be calculated in the same way.

### A novel 18-dimensional natural vector with covariance

After we get the covariance for the pairs of nucleotides, we add the covariance to the original natural vector of the sequence *S*. The number of pairs of nucleotides is ${C}_4^2=6$*.* Thus, the natural vector with covariance of a nucleotide sequences *S* is given as follows:

$$ \left({n}_A,{n}_C,\cdots, {n}_T,{\mu}_A,{\mu}_C,\cdots, {\mu}_T,{D}_2^A,{D}_2^C,\cdots, {D}_2^T, Cov\left(A,C\right)/N, Cov\left(A,G\right)/N,\cdots Cov\left(G,T\right)/N\right). $$

In this study, we utilized the novel 18-dimensional natural vector with covariance to analyze DNA barcodes. This method is alignment-free and does not depend on any assumptions.

### Convex hull

In computational geometry, a convex hull is the smallest convex set containing the points. The points can be in high dimensional space. In this study, we classify DNA barcodes into different species based on the corresponding disjoint convex hulls of 18-dimensional natural vectors of the sequences. Because of the extensive computational time required to directly compute convex hulls in high dimensional spaces, instead of comparing convex hulls directly, we utilized the following method to test whether two high-dimensional convex hulls constructed by two point sets disjoint.

### Linear programming approach

Assume two point sets *A* = {*a*_1_, *a*_2_, ⋯, *a*_*n*_} and *B* = {*b*_1_, *b*_2_, ⋯, *b*_*m*_} in *R*^*k*^ space, the method is to compute whether there are two groups of coefficients *λ*_*i*_ and *μ*_*j*_, 1 ≤ *i* ≤ *n*, 1 ≤ *j* ≤ *m*, such that the following equation holds:

$$ {\sum}_{i=1}^n{\lambda}_i{a}_i={\sum}_{j=1}^m{\mu}_j{b}_j $$*,* here $$ 0\le {\lambda}_i,{\mu}_j\le 1,{\sum}_{i=1}^n{\lambda}_i=1\kern0.5em ,\kern0.5em {\sum}_{j=1}^m{\mu}_j=1 $$

We use the linear programming approach to check if there are two groups of coefficients that satisfy the conditions. If the coefficients exist, then the two convex hulls of the two point sets have interactions, otherwise, the two convex hulls are disjoint [20].

### Linear discriminant analysis

The linear discriminant analysis (LDA) is a generalization of Fisher’s linear discriminant. If two groups are linearly separable, we can use linear discriminant analysis. Linear separable suggests that the groups can be separated by a linear combination of features [21]. This means the two convex hulls of the two point sets have no interaction.

Assume the original data *A* is partitioned into *k* classes as *A* = {*Π*_1_, ⋯*Π*_*k*_} with a set of features $\overrightarrow{x}$, where the *i*th class *Π*_*i*_ contains *n*_*i*_ points and${\sum}_{i=1}^k{n}_i=n$. We try to find a projection matrix to separate two point sets in low dimension where$y={W}^T\overrightarrow{x}$. In the discriminant analysis, two scatter matrices, called with-class (*S*_*ω*_) and between-class (*S*_*b*_) matrices, are defined to quantify the quality of the cluster as follows:

$$ {S}_{\omega }={\sum}_{i=1}^k{\sum}_x\left(x-{m}_i\right){\left(x-{m}_i\right)}^T, $$

$$ {S}_b={\sum}_{i=1}^k{n}_i\left(m-{m}_i\right){\left(m-{m}_i\right)}^T, $$

where ${m}_i=\frac{1}{n_i}{\sum}_{x\in {\varPi}_i}x$ is the mean of the *i*th class, and $m=\frac{1}{n}{\sum}_{i=1}^k{\sum}_{x\in {\varPi}_i}x$ is the global mean.

It is easy to verify that $trace\left({S}_{\omega}^T\right)$ measures the closeness of the vectors within the classes, while$trace\left({S}_b^T\right)$ measures the separation between classes. In the low-dimensional space resulting from the linear transformation *W*, the within-class and between-class matrices become${S}_b^L={W}^T{S}_bW$, and${S}_{\omega}^L={W}^T{S}_{\omega }W$. An optimal transformation *W* would maximize$trace\left({S}_b^T\right)$ and minimize$trace\left({S}_{\omega}^T\right)$. Common optimizations in classical discriminant analysis include:

$$ \underset{W}{\mathit{\max}}\left\{ trace{\left({S}_{\omega}^T\right)}^{-1}{S}_b^T\right\}\ \mathrm{and}\ \underset{W}{\mathit{\min}}\left\{ trace{\left({S}_b^T\right)}^{-1}{S}_{\omega}^T\right\}. $$

The solution can be obtained by applying an eigen-decomposition to the matrix ${S}_{\omega}^{-1}{S}_b$, if *S*_*ω*_ or${S}_b^{-1}{S}_{\omega }$is nonsingular. The reduced dimension by LDA is from one to *k − 1*.

### Random forest

A random forest [22] is a classifier consisting of a collection of tree-structured classifiers {*h*(*x*, *θ*_*k*_), *k* = 1, 2, …}, where the {*θ*_*k*_} are independent identically distributed random vectors and each tree casts a unit vote for the most popular class at input *x*. Specifically, for the *k*th tree, a random vector *θ*_*k*_ is generated independent of the past random vectors *θ*_1_, …, *θ*_*k* − 1_ but with the same distribution. The tree is grown using the training set and *θ*_*k*_, resulting in a classifier *h*(*x*, *θ*_*k*_) where *x* is an input vector. After a large number of trees is generated, they vote for the most popular class. These procedures are called random forests. Bagging algorithm used in random forest helps us improve the stability and accuracy in statistical classification. Out-of-bag error is a value to measure the prediction error rate of bagging algorithm on training dataset in random forest method. It’s can be empirical proved as an unbiased estimation for using a test set of the same size as the training set.

### ROC curve

In this study, the ROC curves are drawn by the following steps. Assume that all the samples belong to *M* classes. Given the number of tree parameter *k* in random forest algorithm, each time one class is considered as positive and the others are regarded as negative. We compute four values for *M* times as follows: (1) the true positive (TP): Number of positive samples predicted correctly; (2) the true negative (TN): Number of negative samples predicted correctly; (3) the false negative (FN): Number of positive samples predicted incorrectly; (4) the false positive (FP): Number of negative samples predicted incorrectly. Then the average of the true positive rate TPR = TP/(TP + FN) and the false positive rate FPR = FP/(FP + TN) for all the *M* classes are calculated. The ROC curve plots TPR as a function of FPR when *k* varies. The AUC value representing the area under the ROC curve (above the *x*-axis) is calculated by the “trapz” function in Matlab software.

## Additional file

Additional file 1: **Figure S1.** Barcode gap analyses for the family *Leotiaceae* using distance histograms. Histograms display the intragenus variation in green and the intergenus variation in blue for the family *Leotiaceae*. **Figure S2.** Barcode gap analyses for the family *Microascaceae* using distance histograms. Histograms display the intragenus variation in green and the intergenus variation in blue for the family *Microascaceae*. **Figure S3.** Barcode gap analyses for the family *Sarcoscyphaceae* using distance histograms. Histograms display the intragenus variation in green and the intergenus variation in blue for the family *Sarcoscyphaceae*. **Figure S4.** Barcode gap analyses for the genus *Epulorhiza* using distance histograms. Histograms display the intraspecific variation in green and the interspecific variation in blue for the genus *Epulorhiza*. **Figure S5.** Barcode gap analyses for the genus *Eremothecium* using distance histograms. Histograms display the intraspecific variation in green and the interspecific variation in blue for the genus *Eremothecium*. **Figure S6.** Barcode gap analyses for the genus *Fomitiporia* using distance histograms. Histograms display the intraspecific variation in green and the interspecific variation in blue for the genus *Fomitiporia*. **Figure S7.** Phylogenetic tree for the genus *Pachyphloeus* with the 12-dimensional natural vector method. **Figure S8.** Phylogenetic tree for the genus *Pachyphloeus* with the 18-dimensional natural vector method. **Figure S9.** Phylogenetic tree for the genus *Pachyphloeus* with the multiple alignment method. **Figure S10.** Phylogenetic tree for the genus *Pachyphloeus* with the k-mer method (k = 5). **Figure S11.** Phylogenetic tree for the genus *Pachyphloeus* with the 14-dimensional natural vector without number feature. **Figure S12.** Phylogenetic tree for the genus *Pachyphloeus* with the 14-dimensional natural vector without mean position feature. **Figure S13.** Phylogenetic tree for the genus *Pachyphloeus* with the 14-dimensional natural vector without normalized variation feature. **Figure S14.** Phylogenetic tree for the genus *Pachyphloeus* with the 12-dimensional natural vector without covariance feature. (DOCX 2782 kb)

## Abbreviations

BOLD: Barcode of Life Data System
CBOL: Consortium for the Barcode of Life
COI: Cytochrome c oxidase
ITS: Internal transcribed spacer
LDA: Linear discriminant analysis
ROC: Receiver operating characteristic curve
