# Supplementary material for: A new efficient method for analyzing fungi species using correlations between nucleotides
Source: BMC Evol Biol. 2018 Dec 27;18:200. doi: 10.1186/s12862-018-1330-y (PMC6307163; doi:10.1186/s12862-018-1330-y)
Supplement: Supplementary file 1 — Figure S1. Barcode gap analyses for the family Leotiaceae using distance histograms. Histograms display the intragenus variation in green and the intergenus variation in blue for the family Leotiaceae. Figure S2. Barcode gap analyses for the family Microascaceae using distance histograms. Histograms display the intragenus variation in green and the intergenus variation in blue for the family Microascaceae. Figure S3. Barcode gap analyses for the family Sarcoscyphaceae using distance histograms. Histograms display the intragenus variation in green and the intergenus variation in blue for the family Sarcoscyphaceae. Figure S4. Barcode gap analyses for the genus Epulorhiza using distance histograms. Histograms display the intraspecific variation in green and the interspecific variation in blue for the genus Epulorhiza. Figure S5. Barcode gap analyses for the genus Eremothecium using distance histograms. Histograms display the intraspecific variation in green and the interspecific variation in blue for the genus Eremothecium. Figure S6. Barcode gap analyses for the genus Fomitiporia using distance histograms. Histograms display the intraspecific variation in green and the interspecific variation in blue for the genus Fomitiporia. Figure S7. Phylogenetic tree for the genus Pachyphloeus with the 12-dimensional natural vector method. Figure S8. Phylogenetic tree for the genus Pachyphloeus with the 18-dimensional natural vector method. Figure S9. Phylogenetic tree for the genus Pachyphloeus with the multiple alignment method. Figure S10. Phylogenetic tree for the genus Pachyphloeus with the k-mer method (k = 5). Figure S11. Phylogenetic tree for the genus Pachyphloeus with the 14-dimensional natural vector without number feature. Figure S12. Phylogenetic tree for the genus Pachyphloeus with the 14-dimensional natural vector without mean position feature. Figure S13. Phylogenetic tree for the genus Pachyphloeus with the 14-dimensional natural vector without normalized variat [file 12862_2018_1330_MOESM1_ESM.docx]

**Additional file**

**Six examples to show significant barcode gap by analyzing genetic distances.**

Figure S1, Figure S2, Figure S3 are for families and Figure S4, Figure S5, Figure S6 are for genera.


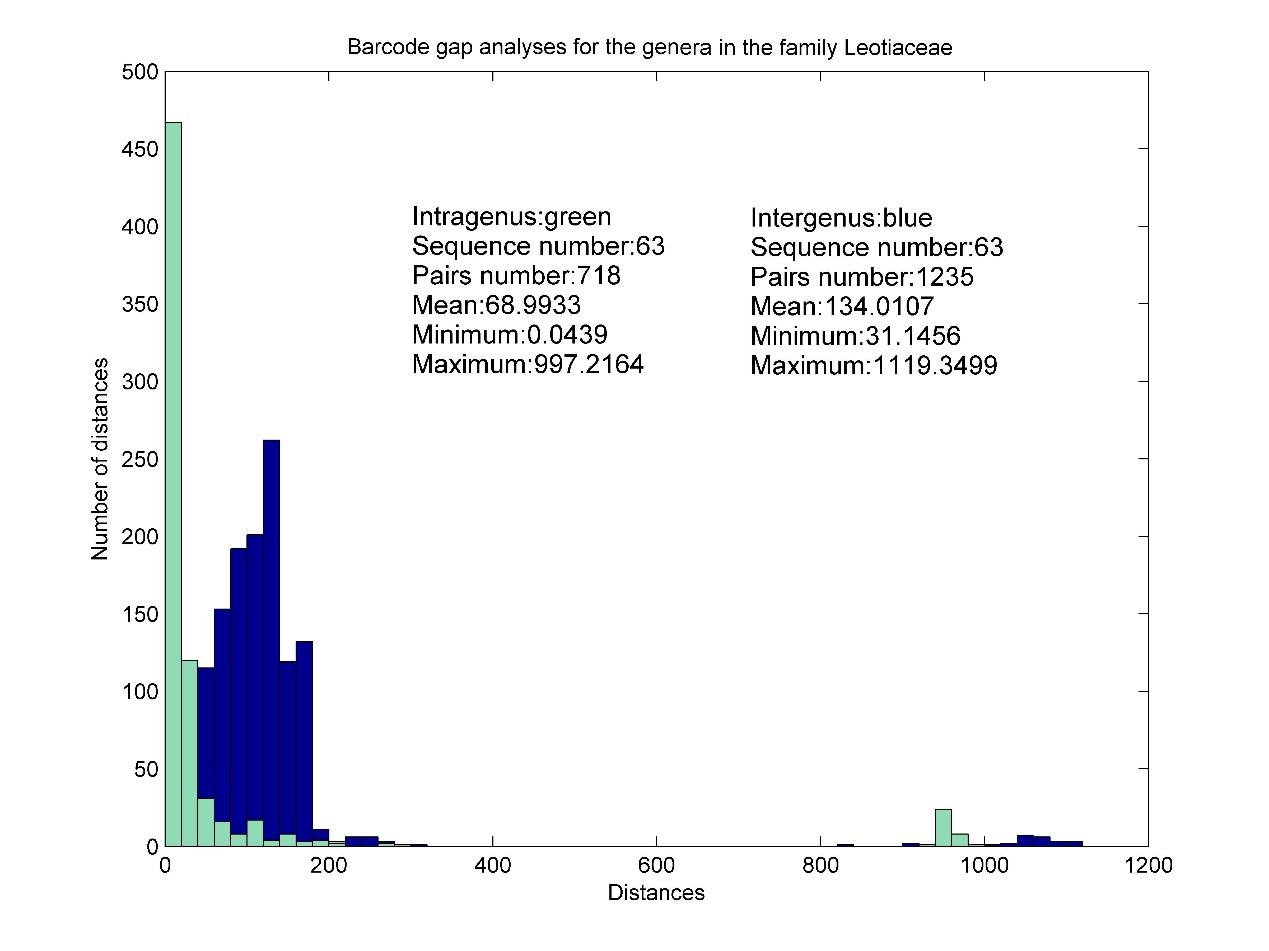


**Figure S1. Barcode gap analyses for the family *Leotiaceae* using distance histograms.** Histograms display the intragenus variation in green and the intergenus variation in blue for the family *Leotiaceae*.


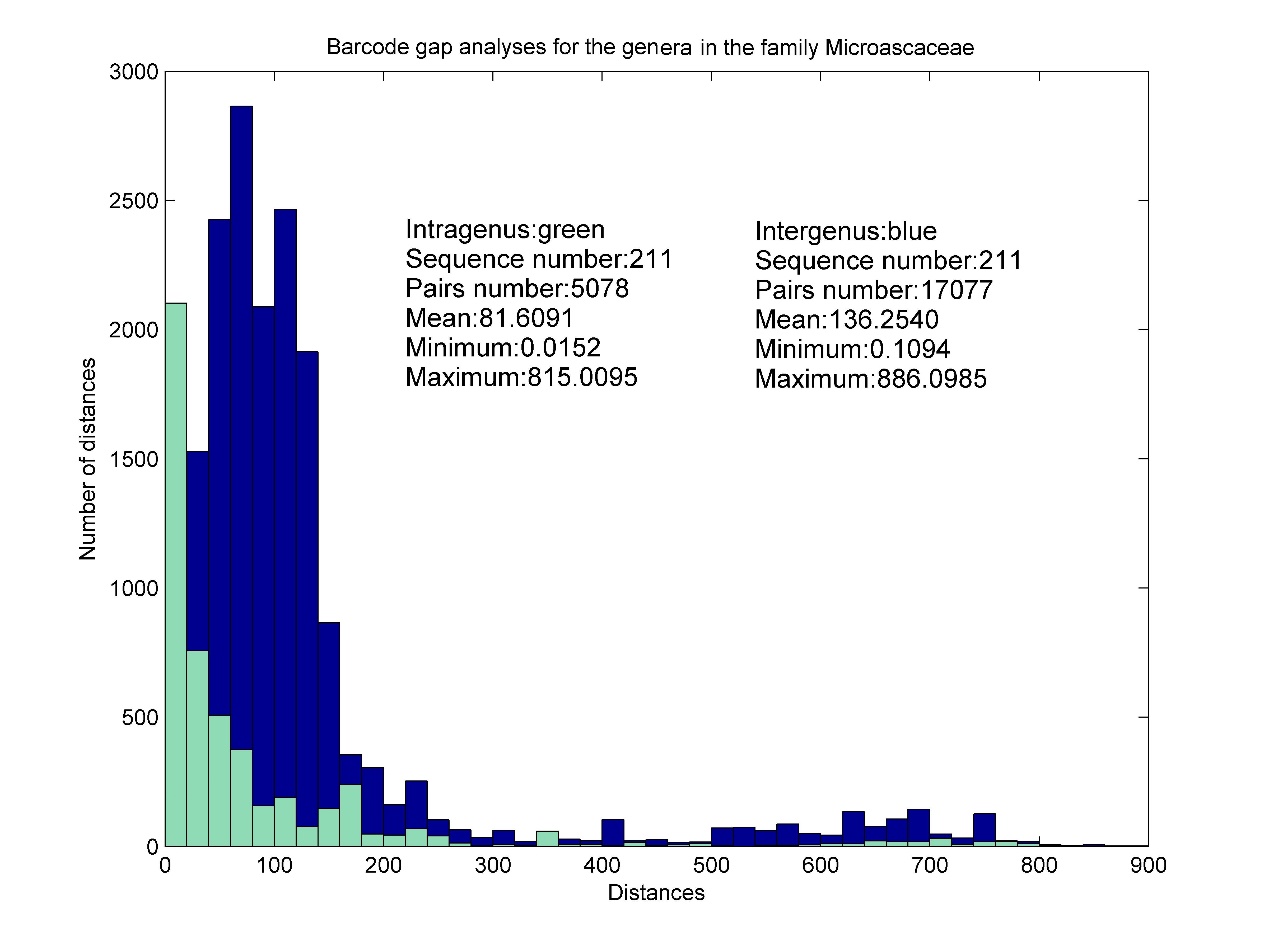


**Figure S2. Barcode gap analyses for the family *Microascaceae* using distance histograms.** Histograms display the intragenus variation in green and the intergenus variation in blue for the family *Microascaceae*.


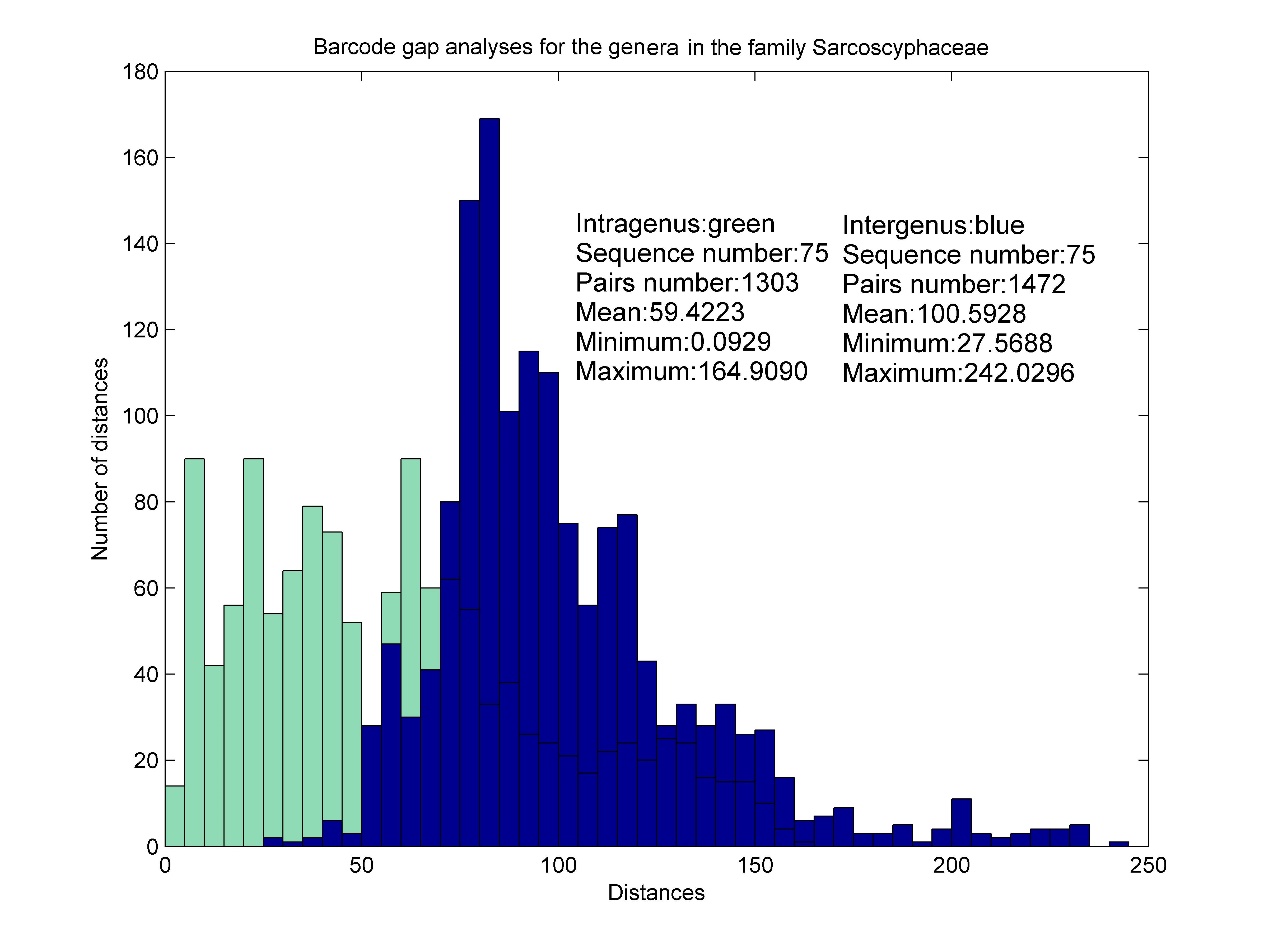


**Figure S3. Barcode gap analyses for the family *Sarcoscyphaceae* using distance histograms.** Histograms display the intragenus variation in green and the intergenus variation in blue for the family *Sarcoscyphaceae*.


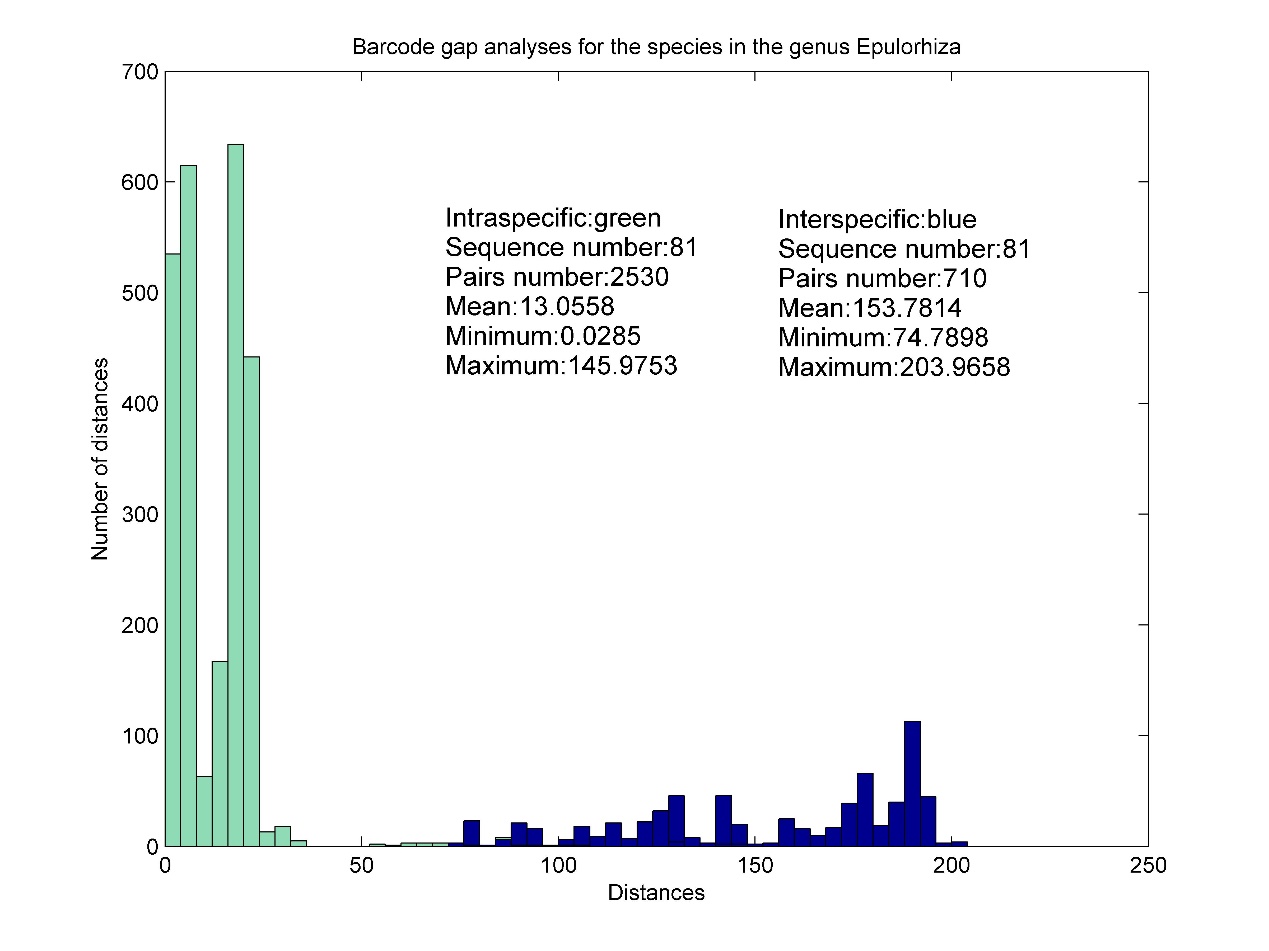


**Figure S4. Barcode gap analyses for the genus *Epulorhiza* using distance histograms.** Histograms display the intraspecific variation in green and the interspecific variation in blue for the genus *Epulorhiza*.


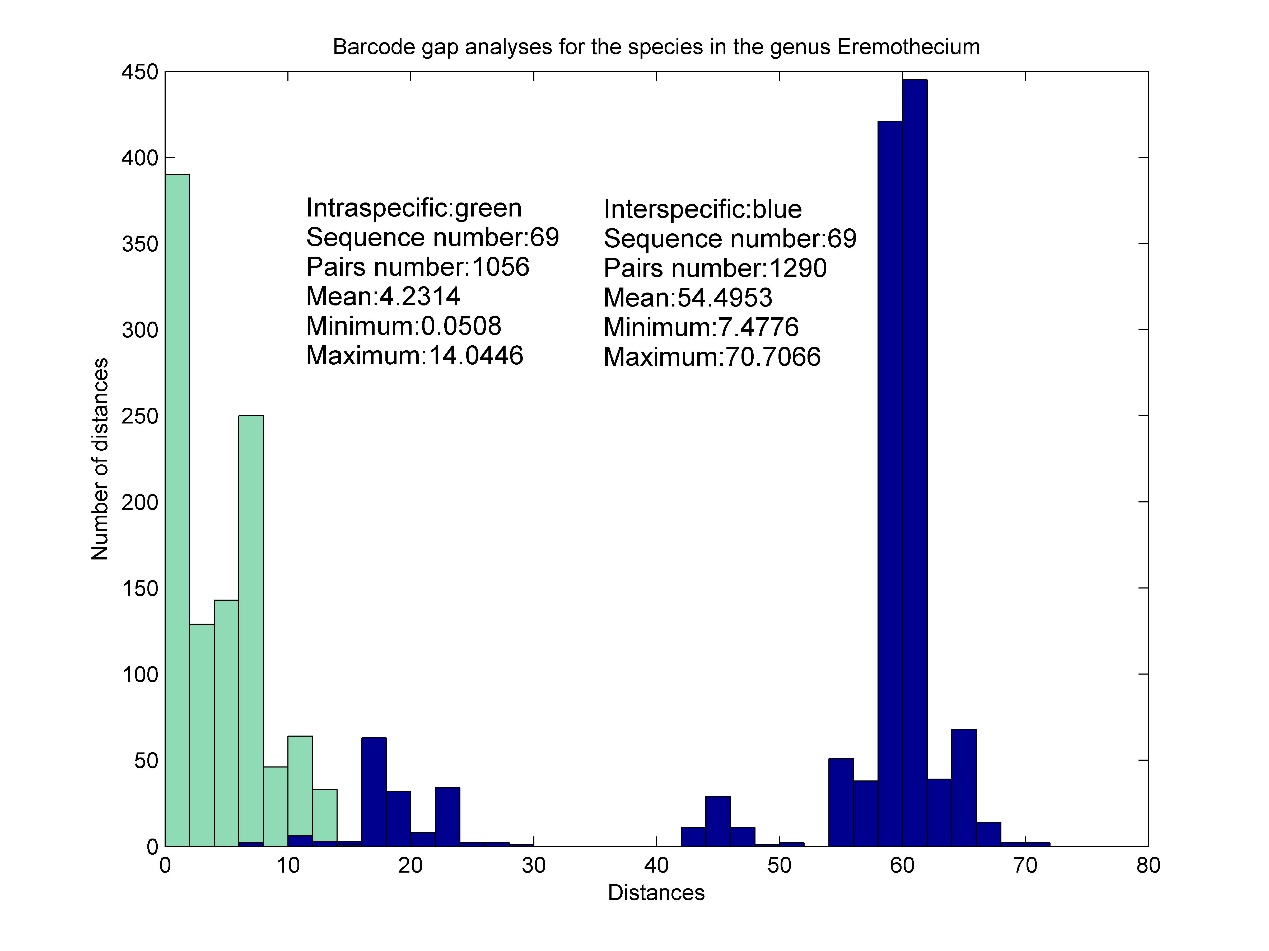


**Figure S5. Barcode gap analyses for the genus *Eremothecium* using distance histograms.** Histograms display the intraspecific variation in green and the interspecific variation in blue for the genus *Eremothecium*.


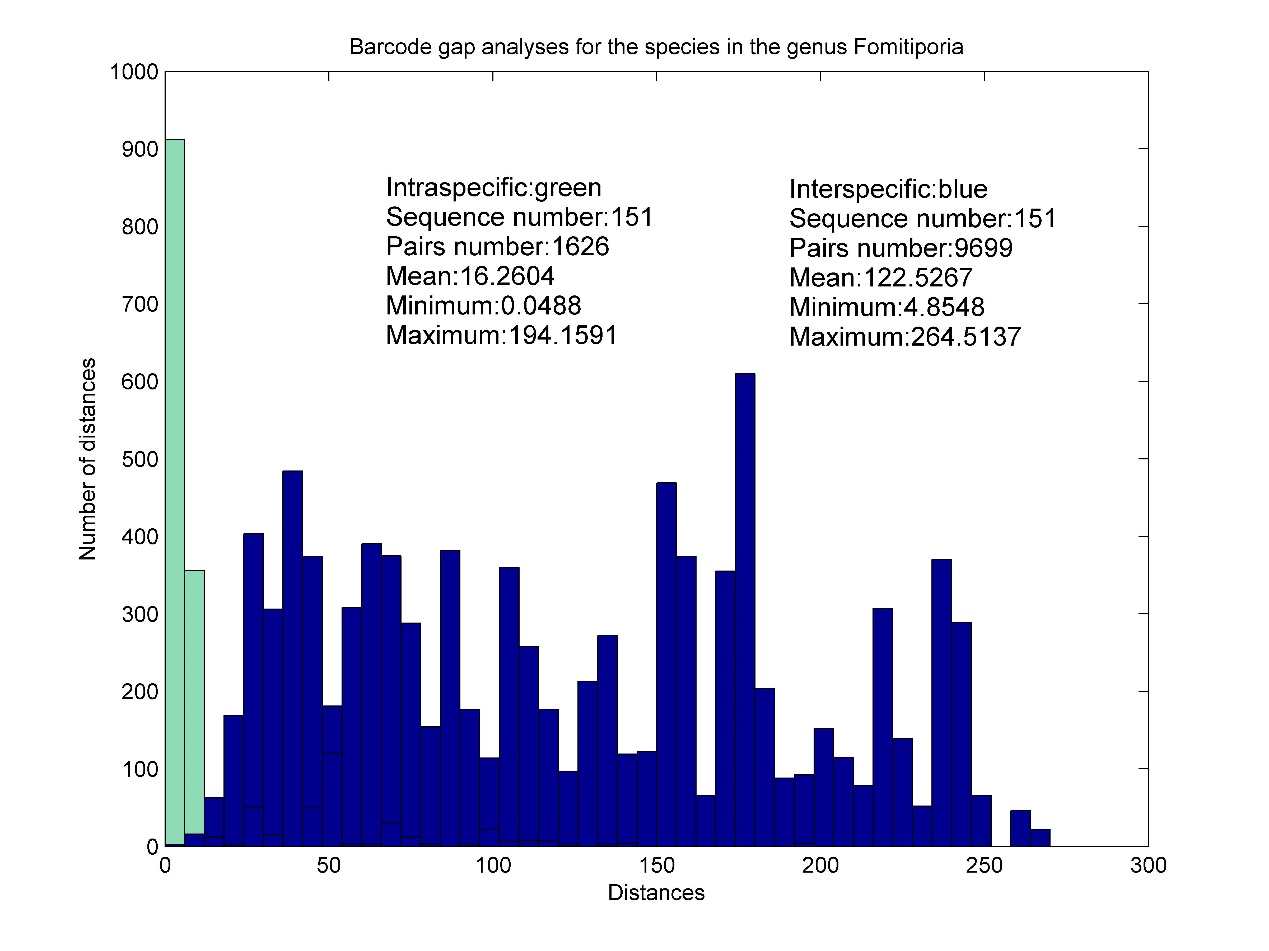


**Figure S6. Barcode gap analyses for the genus *Fomitiporia* using distance histograms.** Histograms display the intraspecific variation in green and the interspecific variation in blue for the genus *Fomitiporia*.

**Phylogenetic analysis--comparison between 18-dimensional natural vector and 12-dimensional natural vector, multiple alignment and k-mer methods**


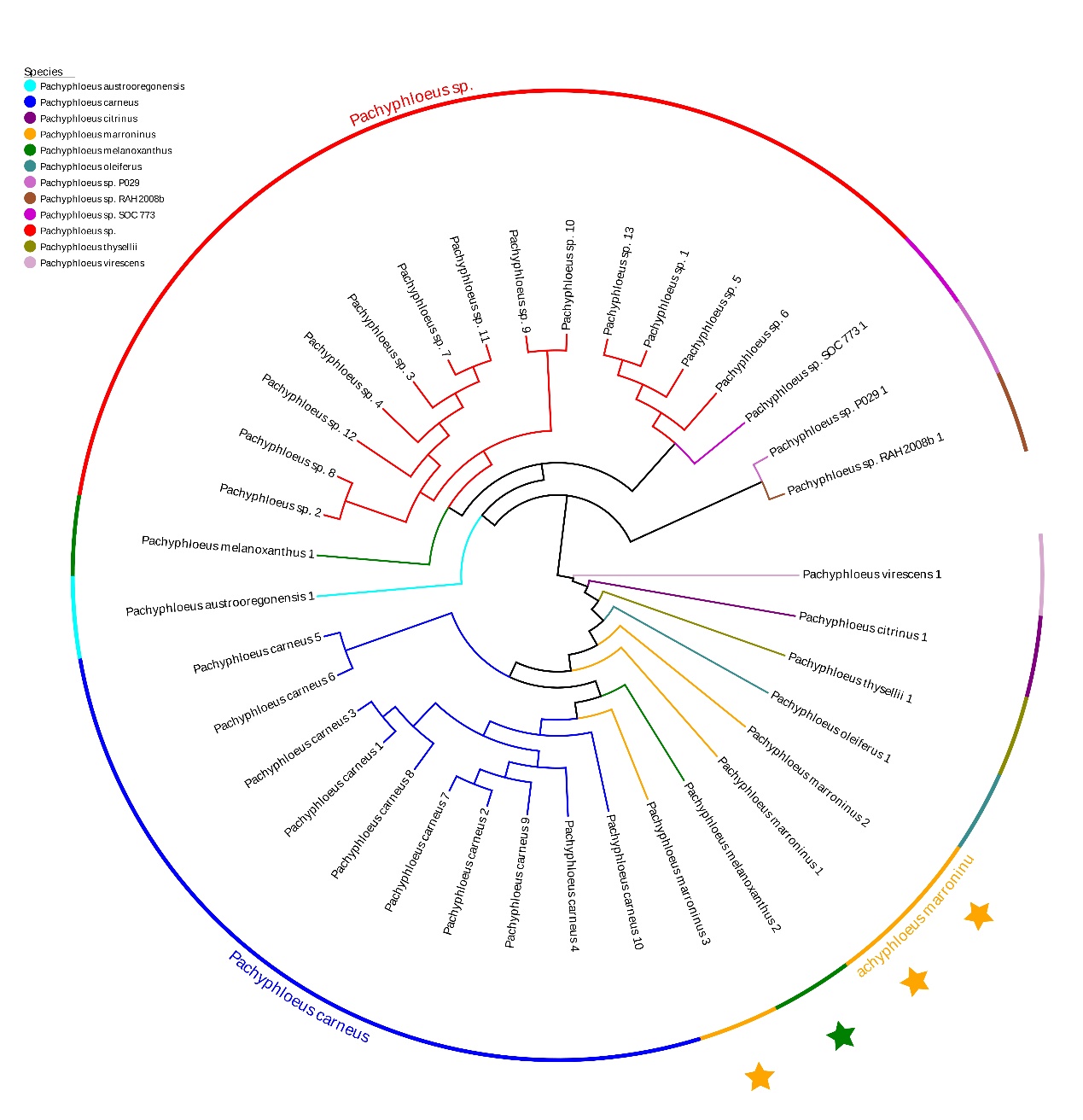


**Figure S7. Phylogenetic tree for the genus *Pachyphloeus* with the 12-dimensional natural vector method.**


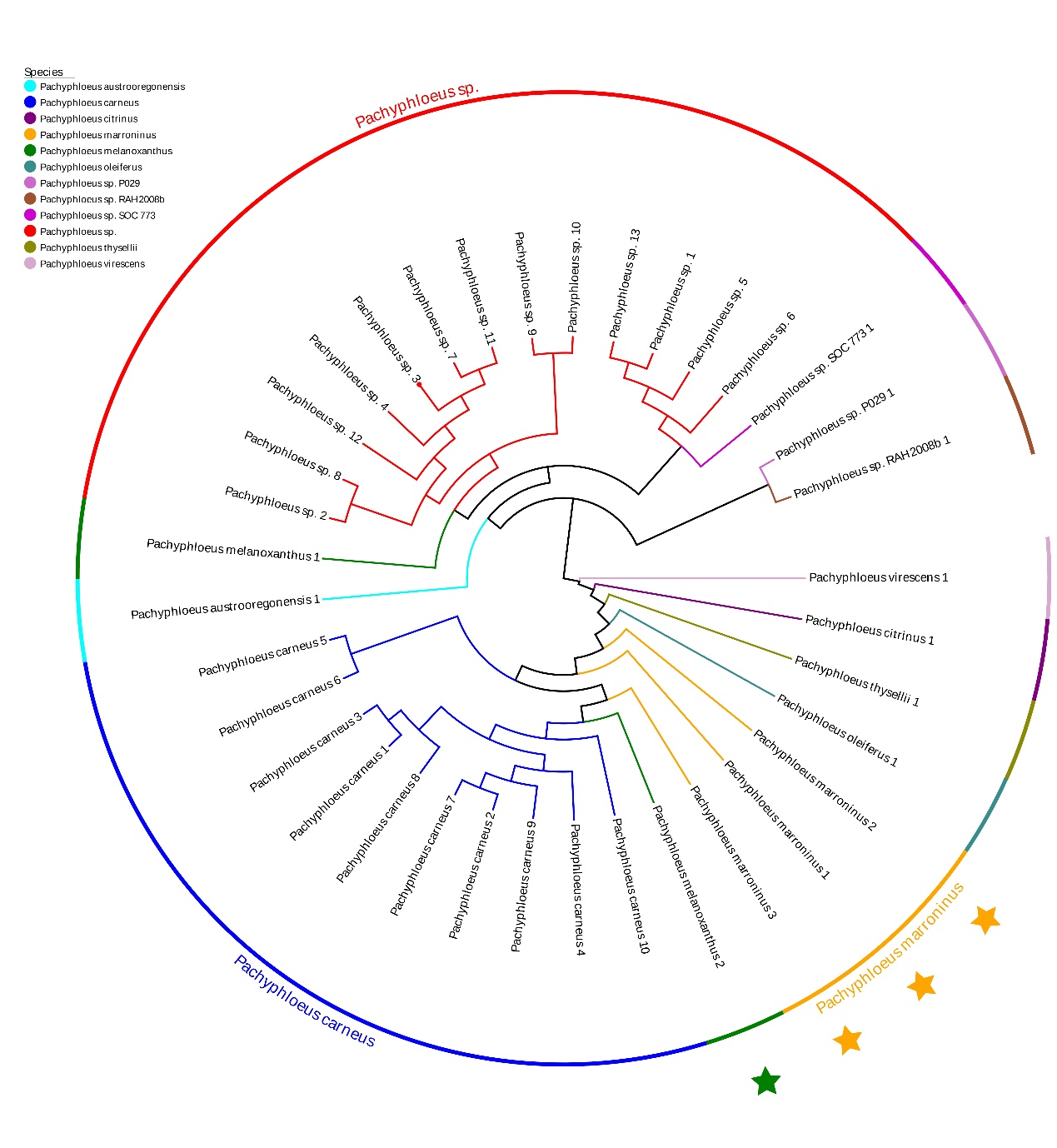


**Figure S8. Phylogenetic tree for the genus *Pachyphloeus* with the 18-dimensional natural vector method.**


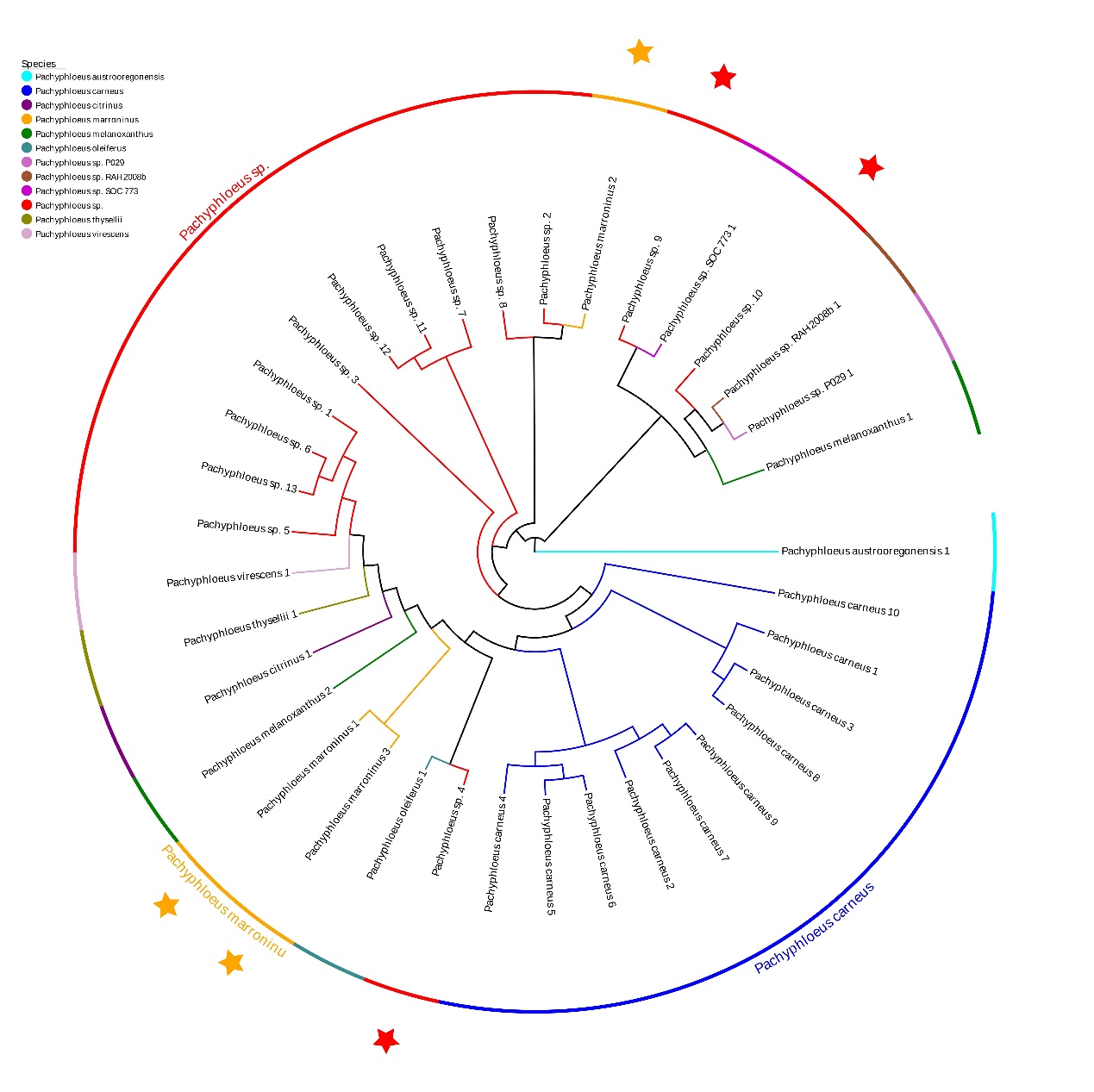


**Figure S9. Phylogenetic tree for the genus *Pachyphloeus* with the multiple alignment method.**


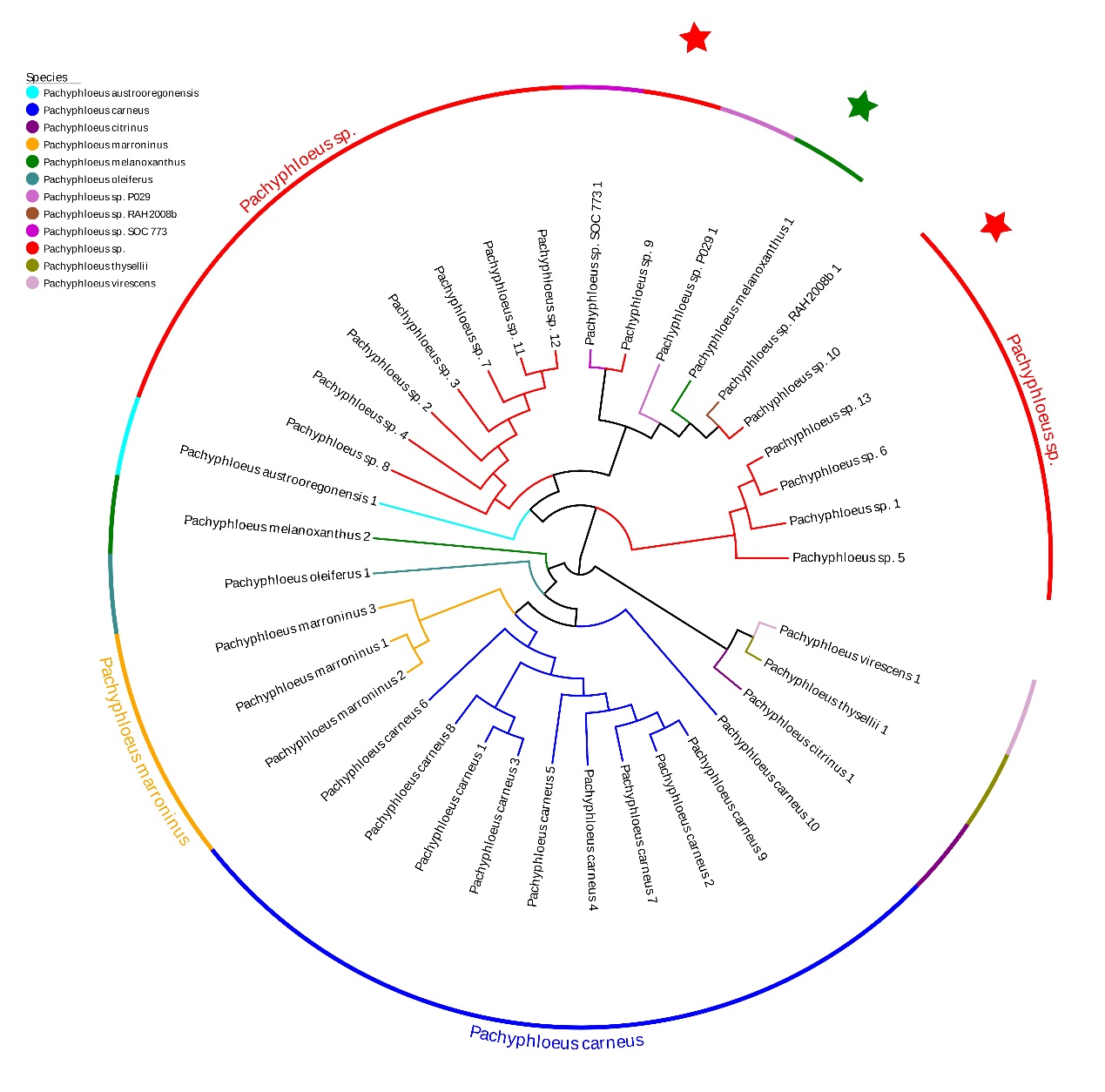


**Figure S10. Phylogenetic tree for the genus *Pachyphloeus* with the k-mer method (k=5).**

**Significance for the four features in 18-dimensional natural vector**


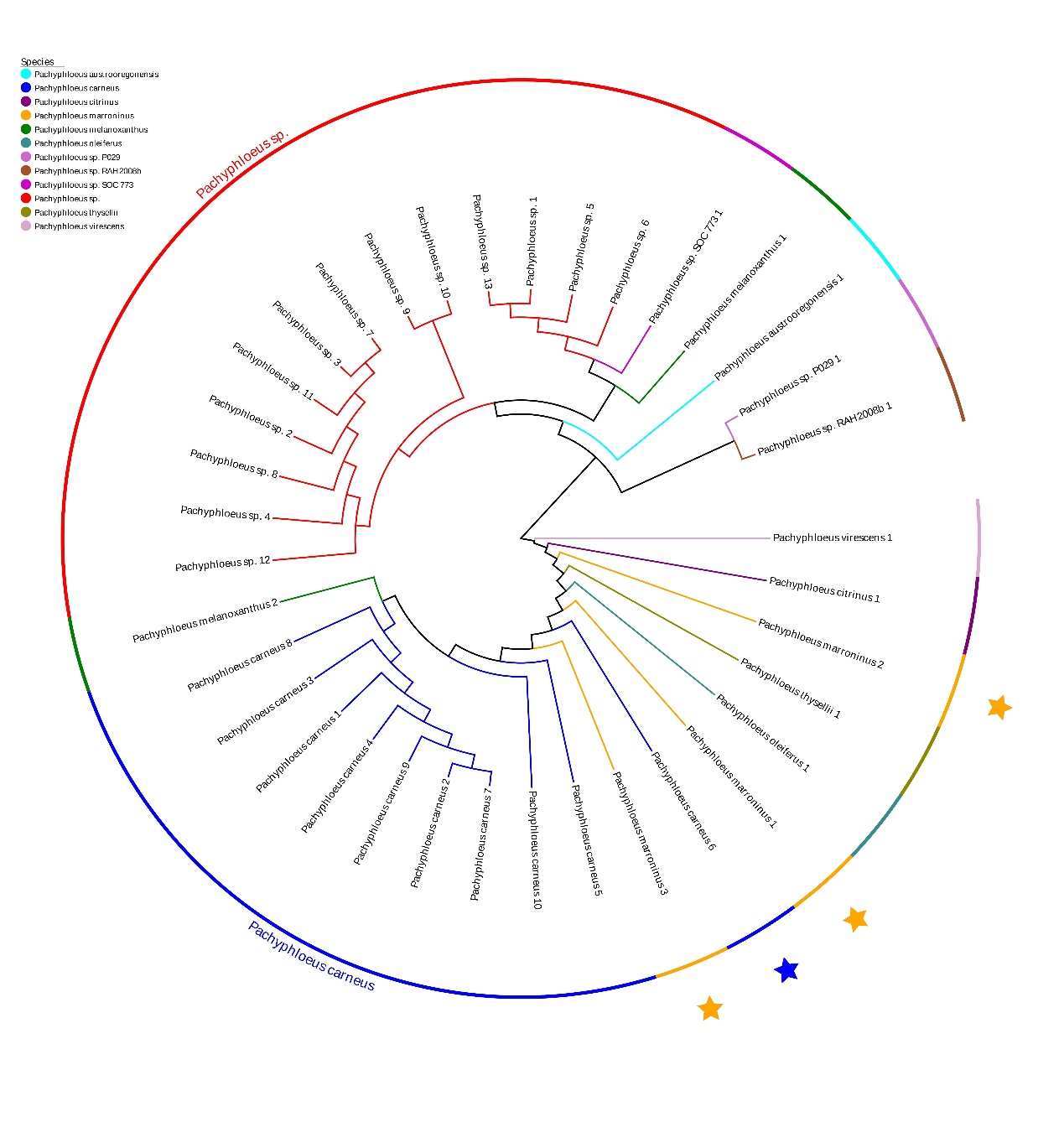


**Figure S11. Phylogenetic tree for the genus *Pachyphloeus* with the 14-dimensional natural vector without number feature.**


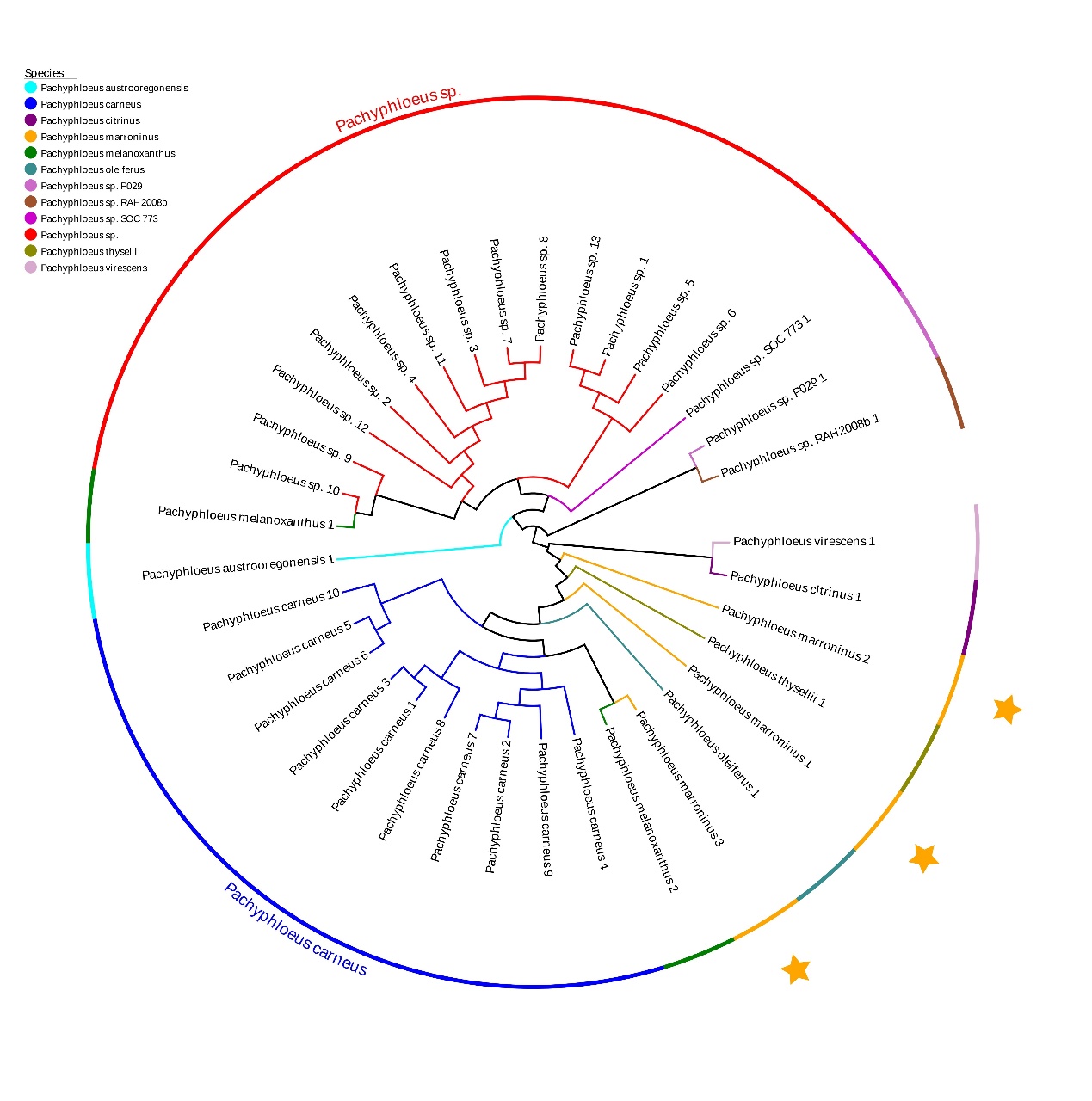


**Figure S12. Phylogenetic tree for the genus *Pachyphloeus* with the 14-dimensional natural vector without mean position feature.**


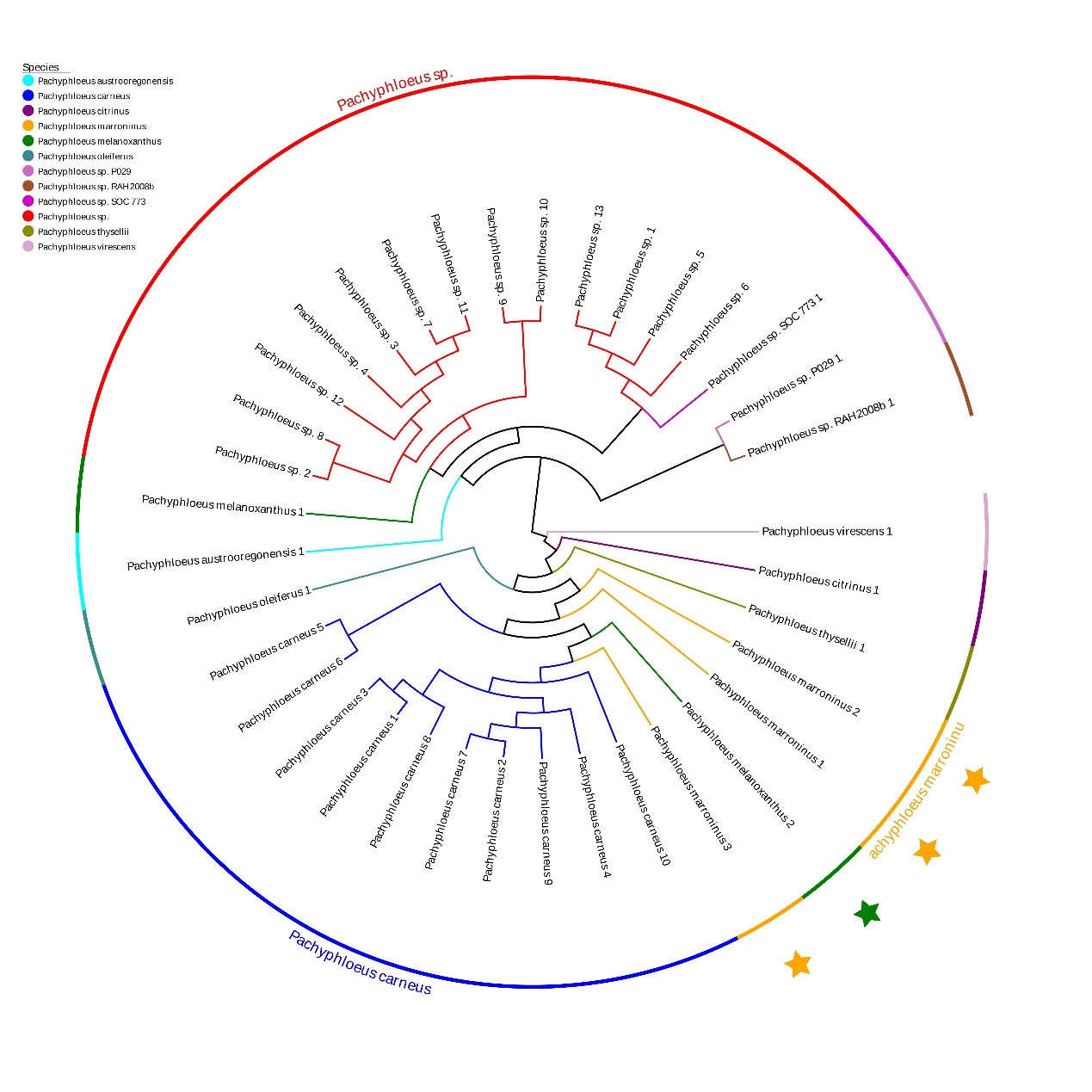


**Figure S13. Phylogenetic tree for the genus *Pachyphloeus* with the 14-dimensional natural vector without normalized variation feature.**


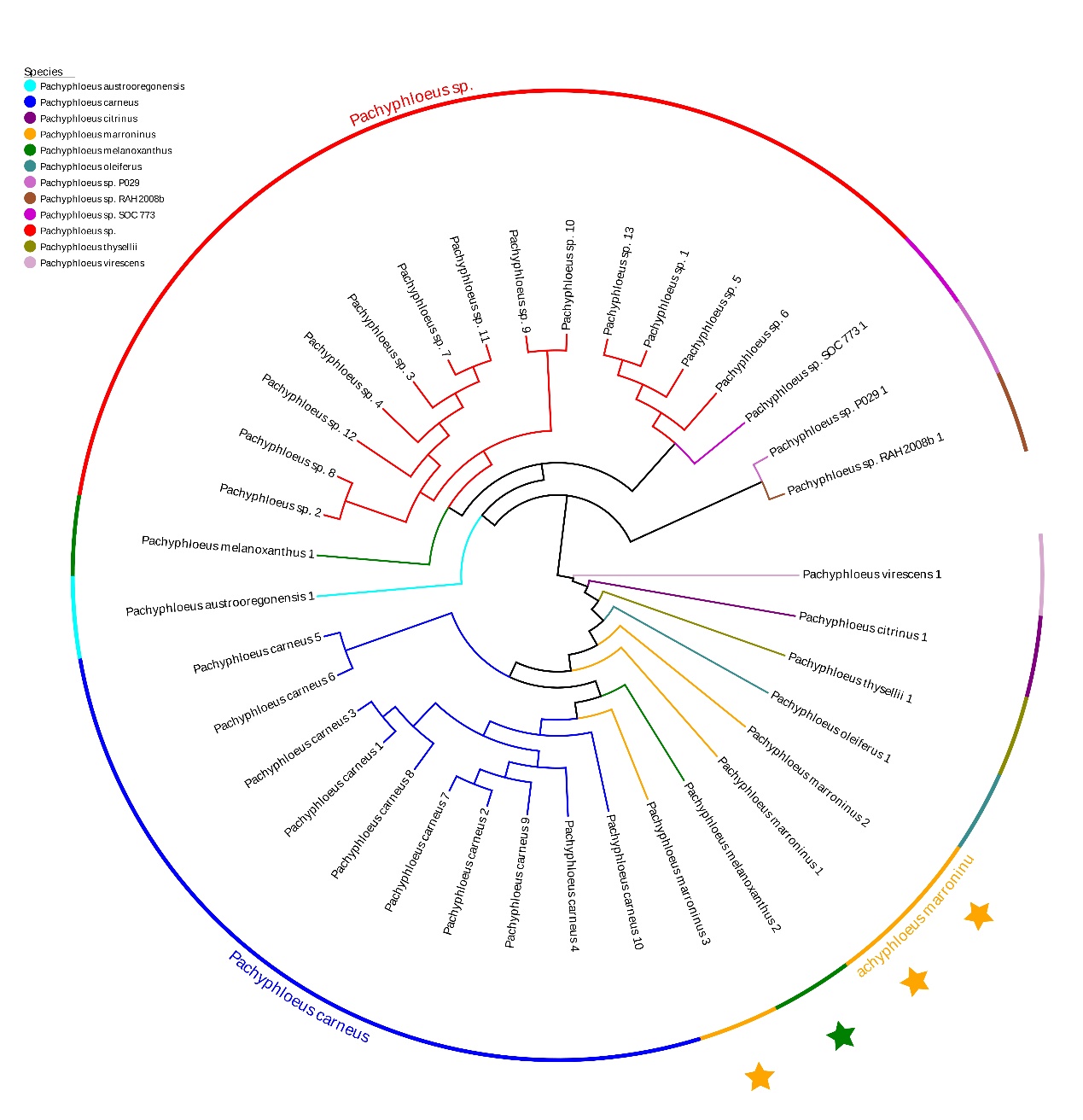


**Figure S14. Phylogenetic tree for the genus *Pachyphloeus* with the 12-dimensional natural vector without covariance feature.**
